# Candidate gene selection and detailed morphological evaluations of *fs8.1*, a quantitative trait locus controlling tomato fruit shape

**DOI:** 10.1093/jxb/erv361

**Published:** 2015-07-14

**Authors:** Liang Sun, Gustavo R. Rodriguez, Josh P. Clevenger, Eudald Illa-Berenguer, Jinshan Lin, Joshua J. Blakeslee, Wenli Liu, Zhangjun Fei, Asela Wijeratne, Tea Meulia, Esther van der Knaap

**Affiliations:** ^1^Department of Horticulture and Crop Science, The Ohio State University/OARDC, Wooster, OH 44691, USA; ^2^Boyce Thompson Institute for Plant Research, Cornell University, Ithaca, New York 14853, USA; ^3^Molecular and Cellular Imaging Center, The Ohio State University/OARDC, Wooster, OH 44691, USA

**Keywords:** Candidate genes, expression analysis, *fs8.1*, genome sequence, morphological analysis, tomato fruit shape.

## Abstract

*fs8.1* is a major quantitative trait locus (QTL) that controls the elongated shape of tomato (*Solanum lycopersicum*) fruit. In this study, we fine-mapped the locus from a 47Mb to a 3.03Mb interval on the long arm of chromosome 8. Of the 122 annotated genes found in the *fs8.1* region, 51 were expressed during floral development and six were differentially expressed in anthesis-stage ovaries in *fs8.1* and wild-type (WT) lines. To identify possible nucleotide polymorphisms that may underlie the fruit shape phenotype, genome sequence analyses between tomato cultivars carrying the mutant and WT allele were conducted. This led to the identification of 158 single-nucleotide polymorphisms (SNPs) and five small indels in the *fs8.1* interval, including 31 that could be associated with changes in gene expression or function. Morphological and histological analyses showed that the effects of *fs8.1* were mainly on reproductive organ elongation by increasing cell number in the proximal–distal direction. Fruit weight was also increased in *fs8.1* compared with WT, which was predominantly attributed to the increased fruit length. By combining the findings from the different analyses, we consider 12 likely candidate genes to underlie *fs8.1*, including *Solyc08g062580* encoding a pentatricopeptide repeat protein, *Solyc08g061560* encoding a putative orthologue of ERECTA, which is known to control fruit morphology and inflorescence architecture in *Arabidopsis*, *Solyc08g061910* encoding a GTL2-like trihelix transcription factor, *Solyc08g061930* encoding a protein that regulates cytokinin degradation, and two genes, *Solyc08g062340* and *Solyc08g062450*, encoding 17.6kDa class II small heat-shock proteins.

## Highlight

We combined fine-mapping, gene expression, genome sequence variation, and morphological and histological analyses to select candidate genes underlying *fs8.1*, a major tomato fruit shape QTL located in a pericentromeric region.

## Introduction

Fruit shape is an important horticultural trait for fruit and vegetable crops. In tomato, fruit shape determines the main use of a particular variety: elongated and blocky fruit are preferred for mechanically harvested processing tomatoes, while fasciated fruit with many locules is ideal for the fresh market and is used for slicing (van der Knaap and Tanksley, 2003; García-Valverde *et al.*, 2013). In addition, fruit shape variation provides a good entry point to investigate the domestication and selection history of tomato, as well as providing fundamental insights into the regulation of plant organ shape (van der Knaap *et al.*, 2014). Based on previous studies, there are two categories of quantitative trait loci (QTLs) that control tomato fruit shape: *lc* (*locule number*) and *fas* (*fasciated*) controlling locule number and ﬂat shape, and *sun*, *ovate*, and *fs8.1* controlling elongated shape (Grandillo *et al.*, 1996; Gonzalo and van der Knaap, 2008; Rodríguez *et al.*, 2011; van der Knaap *et al.*, 2014). Genes underlying tomato fruit shape QTLs were cloned via fine-mapping: *LC* probably encodes an orthologue of WUSCHEL, a homeodomain transcription factor that is required for maintaining the stem-cell identity in the shoot apical meristem (Mayer *et al.*, 1998; Clark, 2001; Muños *et al.*, 2011); *FAS* encodes CLV3, a small secreted protein that acts in the WUS–CLV3 signalling pathway (Schoof *et al.*, 2000; Xu *et al.*, 2015); and *SUN* encodes a member of the IQD family of calmodulin-binding proteins (Xiao *et al.*, 2008). One member of this family, AtIQD1, interacts with both kinesin light-chain-related protein-1 (KLCR1) and calmodulin/calmodulin-like proteins (CaM/CMLs), recruiting them to microtubules (Abel *et al.*, 2005; Bürstenbinder *et al.*, 2013); *OVATE* is the founding member of the OVATE family proteins (OFPs) and its members are often characterized as transcriptional repressors (Liu *et al.*, 2002; Hackbusch *et al.*, 2005; Wang *et al.*, 2007, 2011). However, the gene(s) underlying *fs8.1* has not been identified because reduced recombination rates around the locus prevented the fine-mapping to a short region. This has left a gap in our further understanding of the molecular basis of genes controlling of fruit shape and their putative interactions with each other.

The first report of the *fs8.1* map position was in a BC_1_ population derived from a cross between *Solanum lycopersicum* cv. M82, bearing rectangular elongated fruit, and the closely related wild species *Solanum pimpinellifolium* accession no. LA1589, bearing small round fruit (Grandillo and Tanksley, 1996). The locus *fs8.1* explained up to 27.4% of the fruit shape index (the ratio of height and width) and was further mapped to a 22.8 cM interval on chromosome 8 flanked by markers TG176 and CT92 (Grandillo *et al.*, 1996). With the identification of new recombinants, *fs8.1* was narrowed to a 3.2 cM region flanked by markers CD40 and CT92 (Ku *et al.*, 2000). Single-point analysis conducted in both BC_1_ and BC_2_ populations indicated that CD40 is most closely associated with *fs8.1* (Grandillo *et al.*, 1996), and further studies showed that the locus segregated with a cluster of markers near TG45 (Ku *et al.*, 2000). Unfortunately, the tomato reference genome sequence (The Tomato Genome Consortium, 2012) showed that the 3.2 cM interval was a large region of approximately 47Mb containing ~700 annotated genes. This number far exceeded the chance of identifying the correct candidate gene based on predicted function. In addition to elongated shape, *fs8.1* controls bumpy fruit shape and fruit size in a population derived from a cross with Yellow Stuffer, a tomato variety that features hollow and bumpy fruit similar in morphology to bell peppers (*Capsicum annuum*) (van der Knaap and Tanksley, 2003). These findings suggest that *fs8.1* has pleiotropic effects in controlling distinct aspects of fruit development in different genetic backgrounds. Therefore, *fs8.1* may be useful for breeders to create varieties with a bell and blocky shape as well as elongated fruit shape.

In this study, we sought to identify candidate genes of *FS8.1*. We conducted additional fine-mapping, evaluated the expression of candidate genes, and searched for potential nucleotide polymorphisms that could underlie the mutation leading to fruit elongation. Furthermore, detailed phenotypic evaluations showed that the main effect of *fs8.1* was to uniformly increase cell number in reproductive organs in the proximal–distal direction. This process might be controlled by auxin as its intermediates slightly differentially accumulated in *fs8.1* and WT ovaries. When considering phenotypic, DNA sequence, and expression data as well as the putative function of the genes in the region, several were identified as likely candidates to regulate fruit shape.

## Material and methods

### Plant material, fine-mapping, progeny testing, and near-isogenic line (NIL) development

Two F_3_ plants (08S33-10 and 08S34-10) derived from a cross between *S. lycopersicum* cv. Rio Grande and a wild species, *S. pimpinellifolium* accession no. LA1589, were backcrossed to Rio Grande four times by marker-assisted selection (Supplementary Fig. S1 and Table S1, available at *JXB* online). During the selection, the *fs8.1* locus was maintained in the heterozygous state, while other chromosomes and regions outside the *fs8.1* interval were genotyped to select seedlings that were mostly homozygous for Rio Grande alleles. During the backcross process, recombinant plants were selected for progeny testing to narrow down the *fs8.1* region. Seedlings obtained from these selfed recombinant plants were selected to result in at least five homozygous mutants, five homozygous WT, and two heterozygous at *fs8.1*, and fruit shape was evaluated. The three independent backcrossed and selfed lines used for RNA sequencing (RNA-Seq) were 09S204 (BC_4_F_2_), 09S236 (BC_3_F_2_), and 09S237 (BC_1_F_4_), (Supplementary Fig. S1 and Table S2, available at *JXB* online). The three families used for mapping the *fs8.1* region to 3.03Mb were 11S51 (BC_4_F_2_), 11S65 (BC_4_F_2_), and 11S156 (BC_4_F_3_) (Supplementary Fig. S1). To test whether *fs8.1* segregated in Yellow Pear, an F_2_ population generated from a cross between this variety and LA1589 was evaluated for association of the locus with fruit shape in lines selected to be fixed for *ovate*. Morphological, histological, and auxin metabolism analyses were performed in the BC_4_F_4_, BC_4_F_5_, and BC_4_F_6_ families (13S117, 13S118, 13S161, 13S140, 14S38, 14S92, 14S114, and 14S169), which were considered NILs (Supplementary Fig. S1). The plants used in this study were grown in the field or greenhouse at the Ohio Agricultural Research and Development Center (OARDC, Wooster, OH, USA). To avoid outcrossing, all backcrosses, selfs and recombinant plant seed increases were performed in the greenhouse.

### Single-nucleotide polymorphisms (SNPs) and small indel analysis and marker development

SNPs between *S. lycopersicum* and *S. pimpinellifolium* were identified by comparing their genome contig assemblies at the Sol Genomics Network (SGN, http://solgenomics.net/). Derived cleaved amplified polymorphic sequences (dCAPS) markers were then designed using dCAPS Finder 2.0 (http://helix.wustl.edu/dcaps/dcaps.html) under the option of allowing only one mismatch in the primer. Sequences flanking the high-likelihood indels of 12bp or more were used to design primers using Primer3 (Rozen and Skaletsky, 1999).

For investigating the genomic variation of the 3.03Mb region spanning the *fs8.1* locus between tomato cultivars carrying *fs8.1* and the WT allele, alignments were performed using genome sequences of M82 and Yellow Pear, respectively. The genome sequencing reads of M82 and Yellow Pear were reported by (Bolger *et al.*, 2014*a*) and (Strickler *et al.*, 2014), respectively, and were obtained from SGN FTP site (ftp://ftp.solgenomics.net/genomes/Solanum_lycopersicum/yellow_pear/) and NCBI sequence read archive (http://www.ncbi.nlm.nih.gov/sra/?term=ERA310345 http://www.ncbi.nlm.nih.gov/sra/?term=ERA310345), respectively. The paired-end (PE) reads of both M82 and Yellow Pear were processed using Trimmomatic (Bolger *et al.*, 2014*b*) to remove adaptor and low-quality sequences. Reads shorter than 40bp were discarded. The resulting reads were aligned to the ‘Heinz 1706’ tomato reference genome using BWA (version 0.6.2) (Li and Durbin, 2010). Only one of the duplicated PE reads was kept to minimize the artefacts of PCR amplification, and only reads uniquely mapped (having one single best hit) to the genome were used. Following alignments, SNPs and small indels between Yellow Pear and M82 were identified based on the mpileup files generated by SAMtools (Li *et al.*, 2009). The identified SNPs and small indels were supported by at least two distinct reads.

### RNA-Seq library construction and sequencing

Three partial backcrossed lines were used to analyse gene expression and to identify putative candidate gene(s) underlying *fs8.1* (Supplementary Fig. S1 and Table S2). For each backcrossed line pair, 20 ovaries at the anthesis stage were dissected from five plants each carrying *fs8.1* or the WT allele, respectively, and immediately frozen in liquid nitrogen. Total RNA was extracted with Trizol (Invitrogen, USA) as described by the manufacturer. RNA quantity and quality were assessed using an Agilent Technologies 2100 Bioanalyzer using an Agilent DNA 1000 chip kit. Sequencing was carried out using the Illumina GAII platform (Illumina, San Diego, CA, USA) at the Molecular and Cellular Imaging Center, Wooster, OH, USA. Prior to the library preparation, ~5 μg of total RNA for each samples was treated with RNAase-free DNAase (Invitrogen) to remove the remaining DNA. PE libraries were prepared using an Illumina PE library preparation kit (Illumina) according to manufacturer’s instruction. To select the appropriate size and to remove free adapters, the products were gel isolated on a 2% TAE/agarose gel (Certified Low-Range Ultra Agarose; Biorad). Prior to sequencing, 15 rounds of PCR were performed using the Illumina PE 1.0 and PE 2.0 primers. The library insert sizes were validated using a Bioanalyzer (Agilent Technologies) and quantified using quantitative PCR with a PhiX sequencing control as a standard (Illumina).

### RNA-Seq expression analysis

Filtration, alignment, and calculation of the Illumina reads were carried out according to Huang *et al.* (2013). The final expression data were shown as reads per kb of exon model per million mapped reads (RPKM). Because the six samples came from three independent backcrossed lines, statistical analysis using DESeq could not be performed, and averaging the expression data was also not possible. Thus, we treated the three sets of RNA-Seq data as real-time PCR experiments and analysed the results separately. For further analyses, the RNA-Seq data were filtered using three thresholds: (i) the RPKM value must be >2 in at least two of the three comparisons; (ii) the log2-fold change value (*fs8.1*/WT) must be in the same direction for each comparison; and (iii) as the 09S204 family had the smallest introgression region of the three backcrossed lines (Supplementary Table S3, available at *JXB* online), the absolute value of log_2_ fold change must be >0.5 in the 09S204 family as well as in one of the other two families. To identify the pathway that might be impacted by *fs8.1*, the filtered log2-fold change data were imported into Mapman (http://mapman.gabipd.org/web/guest/mapman) and analysed by aligning against the ITAG2.3 gene annotation (http://mapman.gabipd.org/web/guest/mapmanstore). Genes within the *fs8.1* introgression region were not included in the Mapman analysis.

To identify the candidate gene(s) underlying *fs8.1*, expression of the genes in the 3.03Mb introgression region was investigated in several RNA-Seq datasets including ovaries at anthesis (this study), vegetative and floral meristems in *S. lycopersicum* cv. M82 (Park *et al.*, 2012), and vegetative meristem, young flower bud, and anthesis flowers in *S*. *pimpinellifolium* LA1589 (Huang *et al.*, 2013, http://ted.bti.cornell.edu).

### Morphological and histological analyses

#### Reproductive organ shape analyses 

**Fig. 1.**
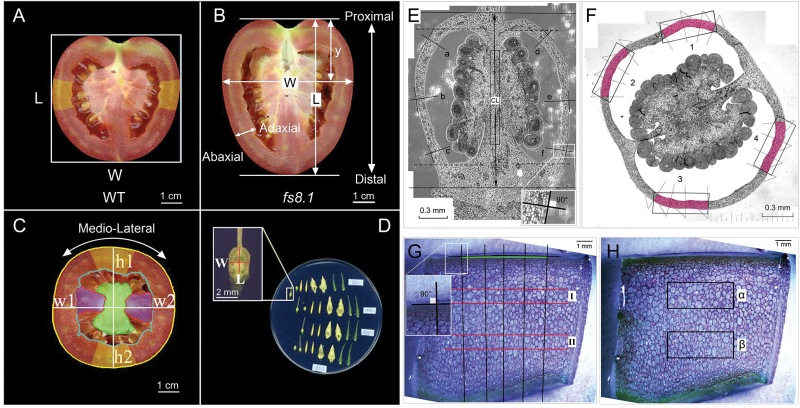
Morphological and histological analyses of reproductive organs in *fs8.1* NILs. (A) Fruit shape index measurement using Tomato Analyzer version 3.0 (TA). Fruit shape index=L/W. (B) Fruit width widest position measurement using Tomato Analyzer. Width widest position=y/L. (C) Fruit structure measurement using Tomato Analyzer. Pericarp thickness=(h1+h2+w1+w2)/4; pericarp area is defined as the area between the yellow and blue lines; the placenta is indicated in green; the septum is indicated in purple. (D) Floral organ shape analysis. Ovary shape index=L/W. (E) Ovary wall and columella cell number and size measurements in a proximal–distal section of an anthesis-stage ovary. CL, columella length; ‘a’ to ‘f’ indicates where the ovary wall thickness and cell layer were measured. (F) Ovary wall cell number measurement in a mediolateral section of an anthesis-stage ovary. ‘1’ to ‘4’ and pink areas indicate where the cell number and size were measured. (G) Cell layer and large-cell size measurements in a pericarp section. The pericarp cell layer was measured along the black lines in the abaxial–adaxial direction. Large-cell size was measured in areas I and II by averaging the areas of the six biggest cells. (H) Average cell size measurement in a pericarp section. Average cell size was measured in two boxes (α and β) at the position of one-third and two-thirds of pericarp thickness; average cell size=area_(α+β)_/total cell number_(α+β)_.

Fully mature tomato fruits collected from families 13S140, 13S117, and 13S118 were cut along the proximal–distal axis and scanned (Fig. 1A, B). The images were opened in Tomato Analyzer version 3.0 (Rodríguez *et al.*, 2010) and morphology parameters were analysed according to the user manual (http://oardc.osu.edu/vanderknaap/tomato_analyzer.php). Floral organs were collected from the 13S117, 13S118, and 13S140 families at anthesis and flattened on 1.5% (w/v) agarose before being scanned (Fig. 1D). Floral organ shapes were evaluated using ImageJ (http://rsbweb.nih.gov/ij/).

#### Fruit and seed weight, and fruit structure analyses 

Fruit and seed weight was evaluated in fruits collected from the greenhouse (13S117 and 13S118) and from field-grown plants (14S92) (Supplementary Fig. S1). Transversely cut and scanned fruit were evaluated for fruit structure, including overall area, and pericarp, placenta, and septum areas using Tomato Analyzer version 3.0 (Fig. 1C).

#### Histological analysis of ovaries at anthesis and mature fruits 

Ultrathin resin-embedded sections of ovary at anthesis and free-hand sectioning of the fresh pericarp were performed in both proximal–distal and mediolateral directions (Fig. 1E–H) (see Supplementary Materials and methods, available at *JXB* online). Ovary sections were collected according to the method of Xiao *et al.* (2009) and imaged using a Leica DM IRB microscope (Leica Microsystems, Germany) coupled to a CCD camera under a ×10 objective lens. The entire ovary was reconstructed using overlapping images and merged with Photoshop CS5 (Adobe, USA), ‘Photomerge’ function, under the option of ‘Reposition’. For mature fruit, free-hand sections were stained with 0.5% toluidine blue in 0.1% sodium carbonate solution (SPI, Electron Microscopy Supplies) and images were taken with a Leica MZFLIII microscope coupled to a digital camera (SPOT RT KE; Diagnostic Instruments). Estimations of cell number and size were performed using ImageJ software.

#### Vegetative organ and flowering time 

Flowering time (defined as leaf number below the second inflorescence), total leaf number (>1cm), leaf length, leaflet number, total internode length, stem thickness, and total side shoot length were determined when the first flower of the second inflorescence had opened. Leaf parameters were measured on the sixth, seventh, and eight leaf. Total internode length and stem thickness were measured from the sixth to the tenth internode. Total side shoot length was investigated for the side shoots that were in the first to fifth leaf axils. Terminal leaflet length, width, and shape index were investigated in the seventh to tenth leaf. Because of the curved shape of the leaflets, they were cut and scanned in pieces, and analysed with ImageJ software. Leaflet shape index was defined as maximal length divided by maximal width.

### Auxin, tryptophan, and tryptamine quantification

Anthesis-stage ovaries of the *fs8.1* NILs (14S38 and 14S114) were collected at 10 a.m. and immediately frozen in liquid nitrogen. Approximately 15–20mg of tissue was weighed and ground in liquid nitrogen and final sample mass was recorded. Samples were extracted with sodium phosphate buffer, pH 7.0, as described by Novák *et al.* (2012), with the following modifications: samples were extracted at 4 °C, and either 25ng of indole-3-propionic acid (Santa Cruz Biotechnology, Dallas, TX, USA) or a combination of 25ng of deuterated d5-indole-3-acetic acid (Olchemim, Czech Republic) and 20ng deuterated d3-tryptophan (C/D/N Isotopes, Quebec Canada) was added as an internal standard. Auxin, tryptophan, and tryptamine were concentrated using solid-phase extraction. Samples were dried under nitrogen gas, redissolved in 1ml of methanol, and passed through a 0.2 μm nylon filter (Fisher Scientific). Auxin, tryptophan, and tryptamine levels were quantified using high-pressure liquid chromatography/tandem mass spectrometry (LC-MS/MS) (Agilent 6460 QQQ LC-MS/MS system) (Novák *et al.*, 2012). One microliter of each sample was injected for LC-MS/MS analyses. Paired Student’s *t*-tests were performed for the statistical analyses.

## Results

### Fine-mapping of *fs8.1* to a 3.03Mb region

**Fig. 2.**
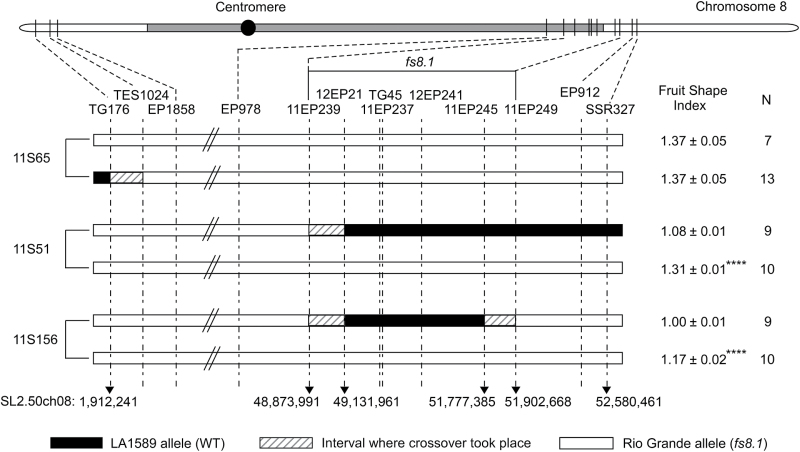
Fine-mapping of *fs8.1* to a 3.03Mb region. Fruit shape index is the ratio of maximal length to maximal width, and eight fruits per plant in each genotype were evaluated; ****P<0.001 by Student’s *t*-test; N, plant number. The positions of the centromere (black dot) and pericentromeric heterochromatin (grey shading) are according to Shearer *et al.* (2014).

Using the cultivated tomato and LA1589 genome sequences for additional marker development, we identified three recombinant plants in the *fs8.1* region. Detailed investigations showed that plant 11S51-18, selfed progeny of plant 10S168#2–51, carried recombination breakpoints between markers 11EP239 at 48.9 and 12EP21 at 49.1Mb on one side and between markers 11EP245 at 51.8 and 11EP249 at 51.9Mb on the other side (Fig. 2). Progeny testing of this line showed a significant association of fruit shape with the allele at the locus (*P*<0.001). Thus, we concluded that *fs8.1* was in the region between markers 11EP239 and 11EP249 with a physical distance of approximately 3.03Mb (Fig. 2). Based on the reference genome and chromosome structure, this region was located on a single scaffold (SGN: SL2.40sc04948) that contained 52 gaps of 100bp or larger (Supplementary Table S4, available at *JXB* online) and might harbour the border of heterochromatin to euchromatin (Shearer *et al.*, 2014).

### Expression analysis of annotated genes in the 3.03Mb introgression

**Fig. 3.**
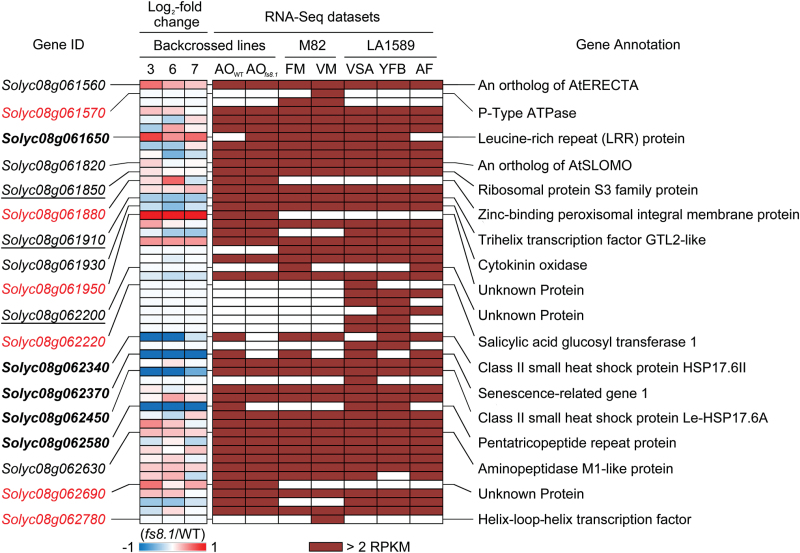
Expression analysis and candidate gene selection in the 3.03Mb introgression. FM, floral meristem; VM, vegetative meristem; VSA, vegetative shoot apex including leaf primordia; YFB, young flower bud; AF, anthesis flower; AO, anthesis-stage ovary; WT, *fs8.1* WT allele; *fs8.1*, *fs8.1* mutant allele. Log_2_ fold change was calculated in the three replicates of ‘Backcrossed lines’ RNA-Seq dataset generated in this study; 3, 6, and 7 indicate families 09S204, 09S236, and 09S237, respectively. In the column ‘Gene ID’, red colour, bold, underline, and regular font, respectively, indicate genes only expressed in VM and AO, differentially expressed genes in AOs of *fs8.1* backcrossed lines, genes harbouring mutations in their putative amino acid sequences, and candidate genes that were selected based on their putative functions. The RNA-Seq datasets of M82 and LA1589 are from Park *et al.* (2012) and Huang *et al.* (2013), respectively.

The gene(s) underlying *fs8.1* may be differentially expressed, thereby causing the fruit shape phenotype. Furthermore, not all annotated genes in this region may be expressed. Therefore, we sought to evaluate gene expression of those that were located in the 3.03Mb region in three RNA-Seq datasets including our own. Of the 122 annotated genes in the introgression region, 51 were expressed at more than 2 RPKM in at least one of the three different datasets (Fig. 3 and Supplementary Table S5, available at *JXB* online). Among the expressed genes, 29 were expressed in all three datasets. Eight genes (*Solyc08g062250*, *Solyc08g062280*, *Solyc08g062310*, *Solyc08g062330*, *Solyc08g062360*, *Solyc08g062220*, *Solyc08g062490*, and *Solyc08g062290*) were only found in the dataset representing tissues from the wild relative, LA1589 (Huang *et al.*, 2013); three genes (*Solyc08g061570*, *Solyc08g06158*, and *Solyc08g062780*) were only found in the dataset representing cultivated tomato M82 meristems (Park *et al.*, 2012); and three genes (*Solyc08g061880*, *Solyc08g061950*, and *Solyc08g062690*) were only expressed in the dataset representing our own RNA-Seq results (Fig. 3, Supplementary Table S5). Four expressed genes (*Solyc08g062340*, *Solyc08g062450*, *Solyc08g062370*, and *Solyc08g062580*) were consistently downregulated (log2-fold change <–0.5) in anthesis-stage ovaries carrying *fs8.1*, whereas two genes (*Solyc08g061650* and *Solyc08g061950*) were upregulated (log_2_-fold change >0.5) (Fig. 3, Supplementary Table S5). These genes encoded members of the class II small heat-shock protein, a senescence-related protein, and a pentatricopeptide repeat-containing protein and leucine-rich repeat (LRR) protein, respectively. In addition to differentially expressed genes, several of the non-differentially expressed genes were related to auxin homeostasis (*Solyc08g061820*), polar auxin transport (*Solyc08g062630*), cytokinin degradation (*Solyc08g061930*), and an ERECTA-like receptor kinase involved in organ shape regulation (*Solyc08g061560*) (Fig. 3, Supplementary Table S5).

### Genetic variations between cultivars carrying *fs8.1* or the WT allele

**Fig. 4.**
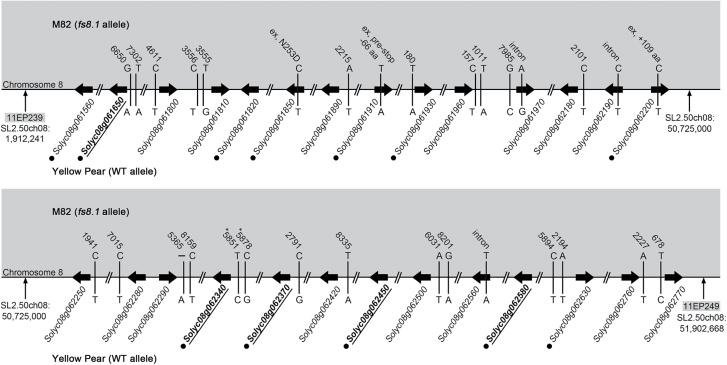
Genome sequence variations between M82 (*fs8.1* allele) and Yellow Pear (WT allele) at the *fs8.1* locus. Black arrows represent expressed genes. Vertical lines represent SNPs or small indels. The number above each SNP/small indel indicates the distance of the SNP/small indel from the nearest gene. Bold font with underlining indicates differentially expressed genes in anthesis-stage ovaries of *fs8.1* backcrossed lines. Black dots indicate high likely candidate genes. –, deletion; ex, exon; *, the SNP was not confirmed in the additional datasets.

To evaluate whether other cultivars carry *fs8.1*, an F_2_ population was generated from a cross between Yellow Pear and LA1589, the latter carrying the WT allele at the locus. As no association of fruit shape with the alleles of *fs8.1* was observed in a population derived from these two parents (Supplementary Table S6, available at *JXB* online), we concluded that the tomato cultivar Yellow Pear carried the WT allele of the *fs8.1* locus. Previously, M82 has been shown to carry the mutant allele of *fs8.1* (Grandillo *et al.*, 1996; Grandillo and Tanksley, 1996; Ku *et al.*, 2000) and therefore M82 and Yellow Pear should differ in the causative nucleotide sequence polymorphism underlying the locus. By aligning the DNA sequences of these two accessions, 158 SNPs and five small indels were identified in the 3.03Mb region spanning the *fs8.1* locus (Supplementary Table S4). Of these, 19 SNPs were located within 10kb upstream of the transcription start point of 14 expressed genes, five SNPs and one small indel were located in 10kb downstream of four expressed genes, and lastly, three SNPs were located in introns of three expressed genes and three other SNPs were located in the exons of three expressed genes (*Solyc08g061850*, *Solyc08g061910*, and *Solyc08g062200*) (Fig. 4, Supplementary Table S4). The three exonic SNPs resulted in a non-synonymous change (N253D), a premature stop codon, and addition of 190 aa, respectively (Fig. 4, Supplementary Table S4). The mutation in *Solyc08g061910* leading to the premature stop codon probably resulted in a null mutation. The high level of genome sequence read depth of 50- to 52-fold and the inclusion of two M82 and Yellow Pear SNP datasets (http://solgenomics.net/jbrowse/JBrowse-1.11.4/?data=data/json/tomato_variants; Lin *et al.*, 2014; E. van der Knaap, unpublished) suggested that these polymorphisms were most likely correct. However, despite the knowledge that structural variation often underlies phenotypic diversity (van der Knaap et al, 2014), large indels, inversions, translocations, and copy number variants were not analysed due to the characteristics of the sequence data.

### Expression level differences suggest that several developmental processes are impacted by *fs8.1*

To determine the effect of the *fs8.1* alleles on gene expression elsewhere in the genome and potential downstream targets, the RNA-Seq data collected from the three different partially backcrossed lines were used to calculate the log_2_-fold change for each expressed gene in each of the lines. After filtering, 607 genes were obtained for enrichment analysis. Using the hypergeometric tests, enriched Gene Ontology (GO) terms for differentially expressed genes were found in the pathways of the cell cycle, cell wall, photosynthesis, and the phenylpropanoid biosynthesis (Supplementary Table S7, available at *JXB* online). Among these, genes involved in the cell cycle and phenylpropanoid biosynthesis (cyclin B and D family proteins, hydroxycinnamoyl CoA quinate transferase,and HXXXD-type acyl-transferase) were downregulated in *fs8.1* compared with WT, whereas genes encoding xyloglucan endotransglycosylase-related proteins involved in cell wall biosynthesis were consistently upregulated in *fs8.1* (Supplementary Table S7). These findings suggested a role for these genes in regulating fruit elongation mediated by *fs8.1*.

### The effect of *fs8.1* is to elongate reproductive organs

**Fig. 5:**
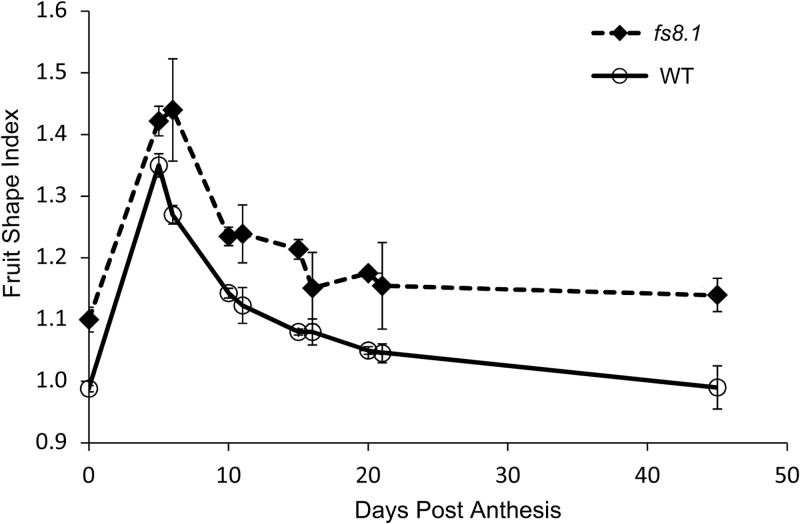
Fruit shape index changes during development. At least three fruits per plant and five plants per genotype were evaluated.

We wanted to know whether *fs8.1* controls the shape of other plant organs in addition to the fruit. The maximal length and shape index were significantly increased in all reproductive organs except for the style, while the maximal width did not differ (Table 1). To evaluate the effect of *fs8.1* on development, shape index changes were monitored during fruit growth. The dynamics of the shape index of *fs8.1* and WT followed the same pattern during fruit development, with the highest value occurring at 5 d post anthesis (Fig. 5). However, the final fruit shape index of both *fs8.1* and WT was similar to the shape index of anthesis-stage ovaries (Fig. 5). In addition to the shape index, *fs8.1* also significantly increased fruit weight and pericarp area. Other traits related to fruit structure and seed weight were not consistently different between *fs8.1* and WT (Supplementary Table S8, available at *JXB* online). For the vegetative traits, only total side shoot length was consistently increased in *fs8.1* and at two different time points (Supplementary Table S9, available at *JXB* online).

**Table 1.** Reproductive organ size and shape index of fs8.1 NILs The two values per organ represent two biological replicates. The upper value was obtained from family 13S140, and lower value was obtained from the 13S117/118 families. *N* indicates the number of plants per genotype. NA, not analysed; NS, not significant. Shape index is the ratio of ‘maximal length’ to ‘maximal width’.

Organ	Maximal length (mm)			Maximal width (mm)			Shape index			*N*
	*fs8.1*	WT	*P value*	*fs8.1*	WT	*P value*	*fs8.1*	WT	*P value*	
Ovary	2.20±0.01	1.93±0.02	8.48E–08	1.96±0.01	1.95±0.01	NS	1.12±0.01	0.99±0.01	4.83E–08	6
	2.12±0.02	1.84±0.02	7.74E–11	1.96±0.03	1.88±0.02	0.03	1.08±0.02	0.98±0.01	4.08E–05	
Fruit	66.93±1.12	59.53±0.84	7.93E–05	60.77±0.99	62.71±0.61	NS	1.10±0.01	0.96±0.01	3.46E–10	8
	69.73±1.00	59.08±0.66	7.80E–07	61.75±0.45	62.18±0.64	NS	1.13±0.01	0.95±0.09	2.42E–08	
Anther	9.08±0.11	8.74±0.06	0.01	1.36±0.01	1.37±0.01	NS	6.68±0.07	6.38±0.05	0.01	6
	9.44±0.09	8.81±0.11	2.00E–04	1.44±0.02	1.39±0.02	NS	6.59±0.09	6.32±0.07	0.03	
Petal	13.03±0.09	12.48±0.11	3.46E–03	4.95±0.08	5.20±0.04	0.01	2.65±0.05	2.41±0.02	2.46E–04	6
	15.60±0.25	13.34±0.20	1.32E–07	6.03±0.21	5.85±0.20	NS	2.62±0.08	2.31±0.07	0.01	
Sepal	14.74±0.42	13.41±0.32	0.02	1.56±0.02	1.58±0.01	NS	9.43±0.19	8.52±0.16	3.26E–03	6
	14.68±0.40	11.90±0.39	3.24E–05	1.64±0.03	1.67±0.03	NS	8.95±0.20	7.14±0.27	1.19E–05	
Style	8.62±0.09	8.51±0.09	NS	0.39±0.01^*a*^	0.42±0.01^*a*^	0.04	NA	NA	NA	6
	8.84±0.20	8.89±0.12	NS	0.45±0.01^*a*^	0.45±0.01^*a*^	NS	NA	NA	NA	

^*a*^ Width was measured at the mid-point of the style.

### *fs8.1* leads to an elongated ovary and fruit by increased cell number in the proximal–distal direction

Changes in organ shape are often accomplished by altered cell division or cell size patterns. To gain further insights into the mechanism by which *fs8.1* leads to elongated ovaries and fruits, we evaluated the cellular parameters at the tissue level (Fig. 1E–H). In both ovaries and mature fruit of *fs8.1*, cell number was significantly increased in the proximal–distal direction in the ovary wall and fruit pericarp, and in the ovary columella (Table 2). In contrast, cell number in the mediolateral direction was not consistently different between *fs8.1* and WT in both ovary and fruit (Table 2). In the abaxial–adaxial direction, *fs8.1* significantly increased the number of cell layers and this was sometimes associated with pericarp thickness (Table 2). With respect to cell size, no significant difference was observed in ovary wall and columella in either the proximal–distal or the mediolateral direction. However, in the mature fruit pericarp, the average cell size in *fs8.1* was decreased in both the proximal–distal and mediolateral direction (Table 2). Thus, the increase in cell number was offset by a decrease in cell size in the *fs8.1* fruit.

**Table 2.** Histological analysis of anthesis ovary and fully grown fruit of fs8.1 NILs For each genotype, six ovaries and 18 fruits from six plants were investigated. The two values per organ represent two biological replicates. The upper value was obtained from family 13S140, and lower value was obtained from the 13S117/118 families. NA, not analysed; NS, not significant.

Parameter	Ovary			Fruit		
	*fs8.1*	WT	*Pvalue*	*fs8.1*	WT	*P value*
Ovary wall/pericarp cell number (proximal–distal direction, 1/2 fruit)	163.33±2.67	135.94±1.00	2.28E–06	499.34±7.09	420.89±13.31	2.08E–04
	169.03±1.41	144.89±2.74	1.44E–05			
Ovary wall/pericarp cell number (mediolateral direction, 1/2 fruit)	227.67±1.27	220.63±0.71	6.93E–04	413.59±2.10	404.16±4.03	NS
	225.58±5.01	226.54±2.94	NS			
Ovary wall/pericarp cell size (proximal–distal direction, 100 µm^2^)	0.1145±0.0431^*b*^	0.1150±0.0261^*b*^	NS	1178.38±42.10^*a*^	1275.56±31.67^*a*^	NS
	0.1030±0.0596^*b*^	0.1039±0.0280^*b*^	NS	864.57±23.74^*b*^	1018.18±31.61^*b*^	3.03E–03
Ovary wall/pericarp cell size (mediolateral direction, 100 µm^2^)	0.0804±0.0680^*b*^	0.0776±0.0950^*b*^	NS	1308.24±34.90^*a*^	1316.85±23.87^*a*^	NS
	0.0768±0.0612^*b*^	0.0727±0.0879^*b*^	NS	954.57±22.83^*b*^	1097.29±28.10^*b*^	2.77E–03
Ovary wall/pericarp cell layer	13.22±0.21	11.80±0.25	1.15E–04	32.50±0.33	27.45±0.42	6.85E–07
	13.77±0.26	12.37±0.08	4.26E–03			
Ovary wall/pericarp thickness (mm)	0.1112±0.0012	0.0983±0.0011	1.10E–05	9.56±0.07	8.77±0.14	3.13E–04
	0.1097±0.0072	0.0999±0.0010	NS			
Ovary columella cell number (proximal–distal direction)	136.78±2.39	114.56±3.16	2.26E–04	NA	NA	NA
	136.20±3.57	125.27±3.07	4.88E–02			
Columella cell size (μm^2^)	125.49±5.84	123.56±6.75	NS	NA	NA	NA
	117.41±11.64	100.11±6.10	NS			

^*a*^ Average size of six largest cells.

^*b*^ Average cell size.

### *fs8.1* decreases tryptophan and tryptamine levels in anthesis-stage ovaries

The hormone auxin is hypothesized to regulate proximal–distal patterning of the pistil and ovary (Nemhauser *et al.*, 2000). Moreover, certain genes within the introgression region have been hypothesized to be involved in auxin metabolism. Therefore, we conducted targeted metabolomic profiling of auxin and auxin metabolites in *fs8.1* and WT ovaries. While indole-3-acetic acid was consistently below the level of detection in this organ, we noticed a decrease in the amount of its precursors, tryptophan and tryptamine, in *fs8.1* ovaries to 88.06 and 81.69% of that of WT, respectively (Supplementary Fig. S2A, B, available at *JXB* online). However, even though this trend was found in five out of six experiments, the values varied among experiments and were barely or not significant, and therefore the results should be interpreted with caution. Interestingly, the ratio of tryptophan to tryptamine was not different between WT and *fs8.1*. This suggested that the conversion of tryptophan to tryptamine was apparently not altered in *fs8.1* (Supplementary Fig. S2C).

## Discussion

### Molecular basis of the *fs8.1* locus

To genetically identify the underlying genes at loci of interest depends on the heritability of the trait, ease of trait evaluation, and recombination frequencies. Centromere and pericentromeric heterochromatin have been shown to be strong suppressors of meiotic recombination (Beadle, 1932; Mather, 1939; Roberts, 1965; Tanksley *et al.*, 1992; Li *et al.*, 2004). Thus, genetically mapping a gene that is located in recombination-suppressed regions will always be a challenge. In this study, we were fortunate to find a rare recombinant plant that narrowed the region to 3.03Mb on a single scaffold that appeared to span heterochromatin and euchromatin. Further recombinant screens failed to identify shorter intervals and therefore we sought to make a list of likely candidates based on expression levels, differential expression, and SNPs and small indels near expressed genes in the 3.03Mb *fs8.1* region. Because the effect of *fs8.1* is clearly final at anthesis (Fig. 5; Ku *et al.*, 2000), genes that were not expressed in anthesis-stage ovaries (our RNA-Seq dataset) but in inflorescence and floral meristems, or in young flower buds, were also likely candidates. Those genes that were only expressed in anthesis ovaries (*Solyc08g061880*, *Solyc08g061950*, and *Solyc08g062690*) or vegetative meristem (*Solyc08g061570*, *Solyc08g062220*, and *Solyc08g062780*) were not likely *fs8.1* candidate genes and were eliminated from the candidate gene list (Fig. 3, Supplementary Table S5). Thus, 45 expressed genes passed and made up the preliminary list of candidates of *fs8.1* (Fig. 3, Supplementary Table S5). As *fs8.1* could be based on a mutation leading to gene expression change, five genes (*Solyc08g061650*, *Solyc08g062340*, *Solyc08g062370*, *Solyc08g062450*, and *Solyc08g062580*) were selected as highly likely candidate genes primarily because of their differential expression between anthesis-stage ovaries carrying *fs8.1* and the WT allele (Fig. 3, Table 3). One of these genes, *Solyc08g061650*, was the only one that was upregulated in anthesis-stage ovaries of *fs8.1*, encoding a LRR domain-containing protein with an unknown function (Fig. 3, Supplementary Table S5). Moreover, two SNPs were identified in the putative promoter region of this gene, which might enhance its expression (Fig. 4, Supplementary Table S4). *Solyc08g062580* encodes a member of the pentatricopeptide repeat protein (PPR) family. The gene was downregulated not only in our *fs8.1* NIL RNA-Seq dataset but also in another RNA-Seq dataset generated from the smallest flower buds of an *fs8.1* NIL in the LA1589 background (Fig. 3, Supplementary Table S5; Y. Wang, S. Wu, and E. van der Knaap, unpublished). For the other three differentially expressed genes, both *Solyc08g062340* and *Solyc08g062450* encoded 17.6kDa class II small heat-shock proteins, whereas *Solyc08g062370* encoded an orthologue of AtSRG1 (Senescence-Related Gene 1). Moreover, a SNP was identified in the putative promoter region of *Solyc08g062370*, which further indicated the potential of this gene to control fruit shape (Fig. 4, Supplementary Table S4). In addition to the candidate genes listed above, mutations leading to amino acid sequence changes could also result in *fs8.1*. Three genes (*Solyc08g061850*, *Solyc08g061910*, and *Solyc08g062200*) were selected as highly likely candidate genes based mainly on the mutations in the coding region (Table 3). *Solyc08g061850* encoded a ribosomal protein S3 harbouring a non-synonymous change from asparagine (N) to aspartic acid (D) at residue 253 (N253D); *Solyc08g061910* encoded an orthologue of potato GTL2-like trihelix transcription factor and harboured a premature stop codon, which would lead to a null mutation; and *Solyc08g062200* encoded an unknown protein and harboured a mutation in the natural stop codon, which could lead to a 109 aa extension of the protein (Fig. 4, Supplementary Table S4).

**Table 3.** Summary of candidate gene selection at fs8.1 locus AO, RNA-Seq dataset of anthesis-stage ovaries of *fs8.1* backcrossed lines (from this study). Upstream indicates that SNPs or small indels are located upstream of the transcriptional start.

Candidate gene	Expressed	Differentially expressed in AO (average of log_2_fold change, *fs8.1/*WT)	SNPs or small indels (compared with WT)	Selected only by putative function (annotation of encoding protein)
*Solyc08g061560*	Yes	No	No	Yes (a putative orthologue of AtERECTA)
*Solyc08g061650*	Yes	Yes (0.60)	Non-coding, upstream^a^	No
*Solyc08g061820*	Yes	No	No	Yes (a putative orthologue of AtSLOMO)
*Solyc08g061850*	Yes	No	Coding, N253D	No
*Solyc08g061910*	Yes	No	Coding, premature stop codon	No
*Solyc08g061930*	Yes	No	Non-coding, upstream	Yes (cytokinin oxidase)
*Solyc08g062200*	Yes	No	Coding, +109aa	No
*Solyc08g062340*	Yes	Yes (–1.26)	Non-coding, upstream^b^	No
*Solyc08g062370*	Yes	Yes (–1.89)	Non-coding, upstream	No
*Solyc08g062450*	Yes	Yes (–1.53)	No	No
*Solyc08g062580*	Yes	Yes (–4.11)	No	No
*Solyc08g062630*	Yes	No	Non-coding, upstream	Yes (aminopeptidase M1-like protein)

^*a*^ A gap was found in the reference genome sequence between the coding region and the SNPs.

^*b*^ SNPs that were not found in both M82 and Yellow Pear genome sequence datasets (Lin *et al.*, 2014; E. van der Knaap, unpublished).

### *fs8.1* represents a different mechanism of regulating tomato fruit shape when compared with other fruit elongation genes

The genes *SUN* and *OVATE* control tomato fruit elongation. Compared with WT, *ovate* primarily increases the fruit proximal end by increasing cell number in the proximal–distal direction and decreasing cell number in the mediolateral direction leading to a pear-shaped fruit (Liu *et al.*, 2002; van der Knaap *et al.*, 2002; Rodríguez *et al.*, 2011; Y. Wang, S. Wu, and E. van der Knaap, unpublished). The *sun* gene, on the other hand, results in long fruit by increasing cell number along the entire proximal–distal direction in the pericarp and columella while decreasing cell number in mediolateral direction in the columella and septum (Wu *et al.*, 2011). The *sun* gene does not alter fruit weight (Wu *et al.*, 2011), whereas *ovate* leads to a slight reduction in fruit weight (Wu *et al.*, 2015). Compared with *sun* and *ovate*, *fs8.1* showed a cellular patterning that was different from the effect of the other two genes: *fs8.1* led to increased fruit shape by increased cell number in the proximal–distal direction without a change in the mediolateral direction (Table 2). This also led to an increase in fruit weight, albeit not always significant (Supplementary Tables S2 and S8). The altered fruit shape was final at anthesis implying that the *fs8.1* pattern is set up during development of the ovary in the flower. This is in contrast to *SUN*, which has been shown to impact ovary shape slightly before anthesis, while the most dramatic shape change occurs immediately after anthesis (van der Knaap and Tanksley, 2001; Xiao *et al.*, 2009; Wu *et al.*, 2015).

Since *fs8.1* appeared to regulate reproductive organ shape by regulating cell number, four genes from the candidate list (*Solyc08g061560*, *Solyc08g061930*, *Solyc08g061820*, and *Solyc08g062630*) could be selected based on their putative functions in the control of organ shape and phytohormones levels (Table 3 and Supplementary Table S5). *Solyc08g061560* encoded a putative orthologue of ERECTA, a LRR receptor-like kinase with functions of regulating cell division, meristem size, fruit morphology, and inflorescence architecture in *Arabidopsis* (Torii *et al.*, 1996; Lease *et al.*, 2001; Shpak *et al.*, 2003; Woodward *et al.*, 2005; Mandel *et al.*, 2014). *Solyc08g061930* encoded a putative orthologue of an *Arabidopsis* cytokinin oxidase, which has been shown to control cytokinin level and regulate reproductive meristem, flower organ, and fruit sizes by impacting cell number in *Arabidopsis* (Bartrina *et al.*, 2011; Köllmer *et al.*, 2014). *Solyc08g061820* encoded a putative orthologue of SLOMO (SLOW MOTION) in *Arabidopsis*, which is an F-box protein involved in the maintenance of a normal plastochron by regulating auxin homeostasis (Lohmann *et al.*, 2010). *Solyc08g062630* encoded a putative orthologue of an *Arabidopsis* aminopeptidase M1-like protein involved in the regulation of auxin polar transport, cell cycle, and seedling development (Peer *et al.*, 2009). Therefore, although there was no evidence at the transcriptional and genome sequence level, these genes were still considered likely candidates for *fs8.1* because of their putative functions.

Of the eight candidate genes that were selected based predominantly on gene expression and genome sequence variations, three of them, according to their putative functions, were deemed the most likely candidate genes. The first one was *Solyc08g062580*, encoding a member of PPR family, which represents a large protein family in land plant species. PPR family members are involved in the regulation of various aspects of plant development by regulating organellar gene expression (Lurin *et al.*, 2004; Petricka *et al.*, 2008; Barkan and Small, 2014). Moreover, a member of the PPR family, At4g18750, has been reported to impact plant morphology, and a knockout of this gene led to a mutant with small size and narrow or pin-shaped leaves, and a disrupted primary vein (Petricka *et al.*, 2008). In addition, *Solyc08g061650* encoded a member of the LRR domain-containing family, which is also a large family that has been implicated in the regulation of a wide variety of developmental and defence-related processes (Torii, 2004; Choi *et al.*, 2011; Torti *et al.*, 2012). The last gene was *Solyc08g061910*, which harboured a probable null mutation and encoded an orthologue of potato GTL2-like trihelix transcription factor. Members of this family in *Arabidopsis* have been reported to regulate perianth architecture, cell growth in trichomes, and responses to cold and salt stresses (Brewer *et al.*, 2004; Breuer *et al.*, 2009; Xi *et al.*, 2012).

In summary and based on the analyses conducted in this study, 12 genes were selected to be the most likely candidates underlying the *fs8.1* locus (Table 3). However, none of them stood out. The gene expression, genome sequence variation, and putative function analyses were performed based on the annotated genes and without evaluation of putative structural variants that often underlie morphological diversity (van der Knaap *et al.*, 2014). Moreover, there are many gaps in the reference genome sequence that may harbour additional genes including *FS8.1* (Supplementary Table S4). Gene action analysis in this and previous studies showed that the WT allele was partial dominant for fruit shape index (D/A=–0.47 in this study), while the *fs8.1* allele was partial or overdominant for fruit weight (D/A=1.14 in this study; Grandillo *et al.*, 1996), suggesting that more than one gene could underlie this locus. Therefore, transgenic complementation using genomic constructs of one, several, or all 12 candidate genes may be required to definitively identify the molecular basis of *fs8.1*. However, as *fs8.1* segregates in cultivated tomatoes (Supplementary Table S6), and with an improved tomato genome sequence underway, association mapping could be employed in the near future to ultimately identify the most likely candidate gene(s) followed by confirmation that includes plant transformations.

## Supplementary data

Supplementary data are available at *JXB* online.

Supplementary materials and methods.

Supplementary Table S1. Markers used in this study.

Supplementary Table S2. Fruit shape index and weight analyses of the three backcrossed lines used in RNA-Seq analysis.

Supplementary Table S3. Progeny test of three backcrossed lines used in RNA-Seq analysis.

Supplementary Table S4. Genome sequence variations between cultivars carrying *fs8.1* and the WT allele.

Supplementary Table S5. Expression and reannotation of genes within the 3.03Mb introgression.

Supplementary Table S6. *fs8.1* segregates in cultivated tomatoes.

Supplementary Table S7. Enrichment analysis of differentially expressed genes in anthesis-stage ovaries of *fs8.1* backcrossed lines.

Supplementary Table S8. Fruit structure and seed analyses.

Supplementary Table S9. Morphological analysis of vegetative organs.

Supplementary Fig. S1. Pedigree map of plant materials used in this study.

Supplementary Fig. S2. Box-and-whisker plot of tryptophan and tryptamine levels in anthesis-stage ovaries of *fs8.1* NILs.

Supplementary Data

## Abbreviations:

LC-MS/MS: pressure liquid chromatography/tandem mass spectrometry
LRR: leucine-rich repeat
NIL: near-isogenic line
PE: paired end
PPR: pentatricopeptide repeat protein
QTL: quantitative trait locus
RNA-Seq: RNA sequencing
RPKM: reads per kb of exon model per million mapped reads
SGN: Sol Genomics Network
SNP: single-nucleotide polymorphism.
