# Supplementary material for: Candidate gene selection and detailed morphological evaluations of fs8.1, a quantitative trait locus controlling tomato fruit shape
Source: J Exp Bot. 2015 Jul 14;66(20):6471–82. doi: 10.1093/jxb/erv361 (PMC4588892; doi:10.1093/jxb/erv361)
Supplement: Supplementary Data [file supp_66_20_6471__index.html]

Candidate gene selection and detailed morphological evaluations of fs8.1, a quantitative trait locus controlling tomato fruit shape — Candidate gene selection and detailed morphological evaluations of fs8.1, a quantitative trait locus controlling tomato fruit shape — Supplementary Data 

# Candidate gene selection and detailed morphological evaluations of *fs8.1*, a quantitative trait locus controlling tomato fruit shape

## Supplementary Data

Data files

- Supplementary Data - Supplementary Data
- Supplementary Data - Supplementary Data
- Supplementary Data - Supplementary Data
- Supplementary Data - Supplementary Data
- Supplementary Data - Supplementary Data
- Supplementary Data - Supplementary Data
- Supplementary Data - Supplementary Data
- Supplementary Data - Supplementary Data
- Supplementary Data - Supplementary Data
- Supplementary Data - Supplementary Data
