# Supplementary material for: Candidate gene selection and detailed morphological evaluations of fs8.1, a quantitative trait locus controlling tomato fruit shape
Source: J Exp Bot. 2015 Jul 14;66(20):6471–82. doi: 10.1093/jxb/erv361 (PMC4588892; doi:10.1093/jxb/erv361)
Supplement: Supplementary Data [file supp_erv361_jexbot151829_file010.pdf]

Candidate gene selection and detailed morphological evaluations of *fs8.1*, a quantitative trait locus controlling tomato fruit shape

Liang Sun, Gustavo R. Rodriguez, Josh P. Clevenger, Eudald Illa-Berenguer, Jinshan Lin, Joshua J. Blakeslee, Wenli Liu, Zhangjun Fei, Asela Wijeratne, Tea Meulia, Esther van der Knaap

Supplementary material and method

Ultra-thin resin sections of anthesis-stage ovaries

Anthesis-stage ovaries were collected and immediately immersed in a solution composed by 3% (v/v) glutaraldehyde, 2% (v/v) paraformaldehyde in 0.1 M potassium phosphate buffer, pH 7.2 for fixation. Samples were placed in a vacuum chamber for infiltration for 4 h, and then were placed on a tissue rocker overnight at room temperature. Ovaries were washed three times with 0.1 M potassium phosphate buffer (pH 7.2), 15 min each, and then were washed three times by double distillated water, 15 min each. Next, ovaries were successively immersed in a series of gradient ethanol-water solutions (25%, 50%, 70%, 90% and 100%, v/v) for dehydration, 30 min for each solution and 3 times in 100% ethanol. The dehydrated ovaries were in infiltrated with a series of gradient LR White-ethanol solutions (50%, 70% and 100%, v/v) (LR White, Electron Microscopy Scinences Co., Cat. #14383-CAT), 1 h for 50% and 70% solutions respectively and repeat infiltration four times in 100% LR-White. Then ovaries were embedded in capsules with 100% LR White and then incubated 24 h at 55°C for hardening. Before sectioning, sample blocks needed to be orientated to ensure that the proximal-distal or medio-lateral axis of the ovary is parallel with the microtome blade. Then, 1 µm thickness section in proximal-distal or medio-lateral direction was obtained from a Leica EM UC6 ultra microtome (Leica Microsystems, Germany). The ovary sections then were stained with a solution of 0.1% (w/v) Na_2CO_3 and 0.5% (w/v) Toluidine blue in 25% ethanol.

Cell number and size analyses of anthesis-stage ovaries

Through merged ovary section images, several ovary attributes were evaluated: ovary wall cell number and cell size in both proximal-distal and medio-lateral direction, ovary wall cell layer, ovary wall thickness and columella cell number and size in proximal-distal. All the measurements in this experiment were performed using imageJ software. Ovary wall thickness and cell layer were measured at six positions along with the lines in the abaxial-adaxial direction (Fig. 1E). By counting the cell number in the 2nd, 6th and 10th layers which were numbered in abaxial-adaxial direction, ovary wall cell number in the proximal-distal direction was investigated in both left and right side of ovary wall, which was defined as the area between the solid lines tangent to the top or the bottom of the ovary. Ovary wall cell number in the medio-lateral direction was counted in the entire ovary wall cycle in the 2nd, 6th and 10th layers that were numbered in abaxial-adaxial direction. For a more conventional comparison, ovary wall cell number in proximal-distal direction was represented in the average value of both left and right side and the cell number in medio-lateral direction was represented in the half cycle value of the ovary wall. Ovary wall cell size in the proximal-distal direction was calculated by dividing the ovary wall area value (area between the two broken lines) by the total cell number within that area. Ovary wall cell size in the medio-lateral direction was investigated in four regions of each section (Fig. 1F, four solid line boxes). Within each region, cell size was calculated by dividing the area value (magenta) by total cell number within that area. Columella in this study was defined as the middle area near proximal-distal axis and between the two solid lines which were perpendicular to the proximal-distal axis and tangent to the top and bottom of the ovary. The bottom of the ovary was defined as a straight line between the two intersections of the ovary with the petal. Columella cell size was calculated by dividing the area of the box (Fig. 1E) by the cell number with the box. For this experiment, two sections per each ovary, six ovaries per genotype were investigated and the whole experiment was repeated twice.

Free-hand sectioning of the fresh pericarp

Tomato fruits were collected at breaker stage and then free-hand sectioning (about 0.1 cm × 1.5 cm × pericarp thickness) of the fresh pericarp was performed in both proximal-distal and

medio-lateral directions. The fruit areas where sections had been cut are indicated with yellow in Fig. S1A and C. To visualize the cells, sections were stained with 0.5% Toluidine Blue in 0.1% sodium carbonate solution (SPI, Electron Microscopy Supplies), and photographed with a Leica MZFLIII dissecting microscope coupled to a digital camera (SPOT RT KE, Diagnostic Instruments).

Cell number and size analysis of pericarp section

Auxiliary lines and boxes were added to the images via Photoshop CS5 (Adobe, USA) and ImageJ software was employed to measure the cell size and number. Pericarp thickness and cell layer were measured and counted respectively along the five lines in abaxial-adaxial direction (Fig. 1G). Pericarp cell number in proximal-distal and medio-lateral direction was calculated by multiplying the pericarp perimeter by the cell number corresponding to 1 mm pericarp perimeter. The pericarp perimeter was measured using Tomato Analyzer v3.0. Cell number corresponding to 1 mm pericarp perimeter was investigated at 1/3 and 2/3 positon (between the red lines "I" and "II" shown in Fig. 1G) in abaxial-adaxial direction of each section and the value was calculated by dividing the length of pericarp surface (green line in Fig. 1G, measured with ImageJ) between the first and fifth line which were perpendicular to the pericarp surface by the average cell number in the direction of abaxial surface of the pericarp (between the red lines shown in Fig. 1G). For comparison with the ovary wall cell number, pericarp cell number in both directions was represented as the half value of entire pericarp. Pericarp cell size in the sections of both proximal-distal and medio-lateral direction was measured in two regions (two boxes shown in Fig. 1H, the boxes were drawn at 1/3 and 2/3 of pericarp thickness). In order to measure the cell size more accurate, two methods were employed. In the first method cell size was calculated by dividing the box area by the total cell number within the box. And in the second method, six largest cells at 1/3 (box_{α}) and 2/3 (box_{β}) position (red lines shown in Fig. 1G) were measured using imageJ software and average cell size = $area_{(\alpha+\beta)}$/total cell $number_{(\alpha+\beta)}$. For all the counting and measuring, at least three sections per each fruit were made and at least three fruits per each plant, five plants per each genotype were investigated.

Homolog gene identification and gene re-annotation

We performed a manual re-annotation of all the annotated genes within the 3.03 Mb introgression region. First, for each tomato gene, the cDNA sequence was obtained from the SGN (http://solgenomics.net/) and then loaded into NCBI ORF Finder (http://www.ncbi.nlm.nih.gov/gorf/gorf.html) to identify the open reading frame (ORF). After that, the deduced amino acid sequence was used to BLAST against the Arabidopsis amino acid database in TAIR (https://www.arabidopsis.org/). Finally, the Arabidopsis gene of which the putative amino acid has the highest similarity to the query amino acid was selected as the putative orthologous gene. If TAIR BLAST result returns to a very low similarity (< 20%) or a very low coverage (< 20%), then the putative amino acid sequence will be BLAST in NCBI (http://www.ncbi.nlm.nih.gov/), and the homolog gene and re-annotation will be obtained from other species, such as potato (*Solanum tuberosum*) and tobacco (*Nicotiana tomentosiformis*).

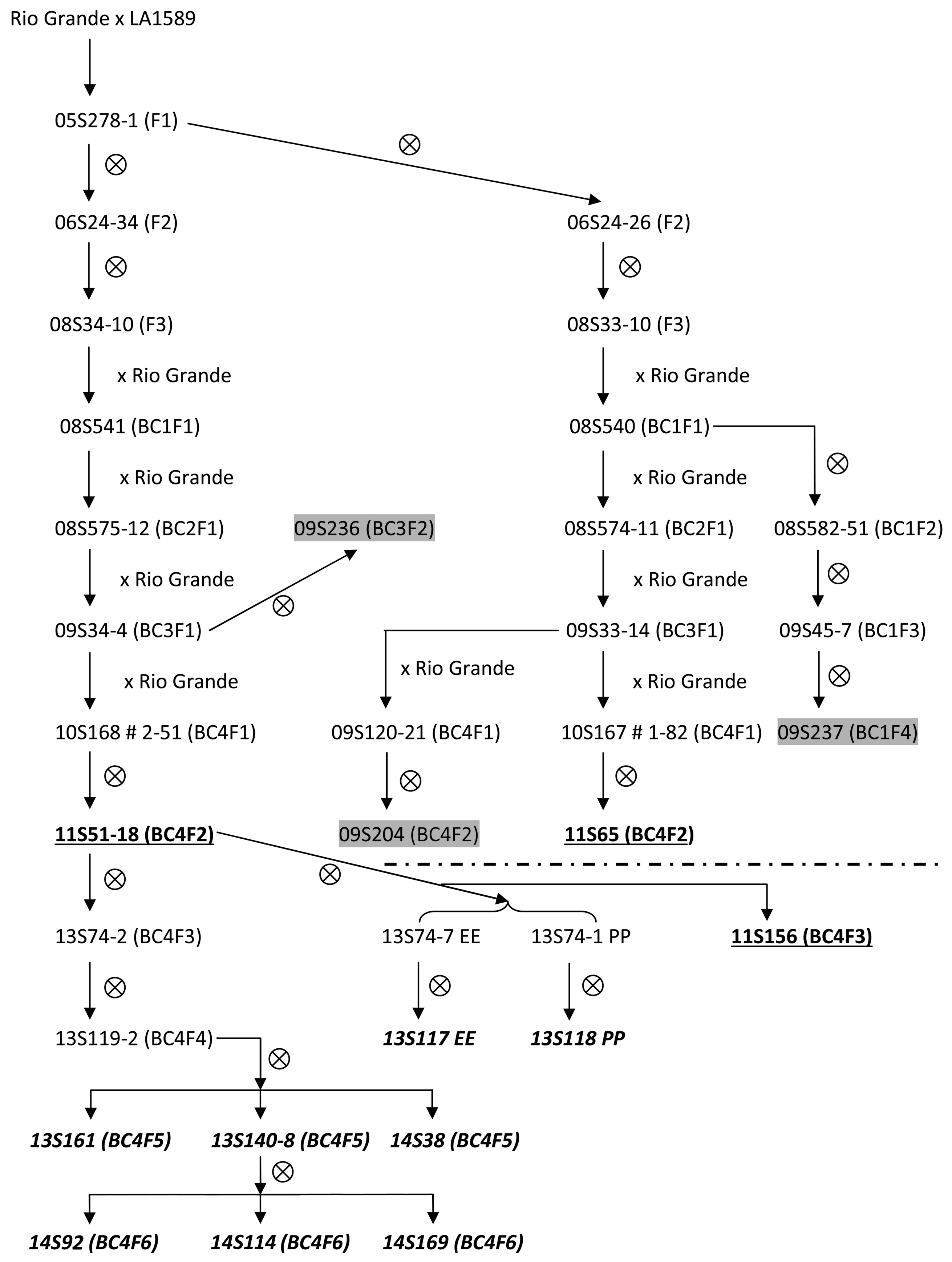
Rio Grande x LA1589
05S278-1 (F1)
06S24-34 (F2)
06S24-26 (F2)
08S34-10 (F3)
08S33-10 (F3)
x Rio Grande
x Rio Grande
08S541 (BC1F1)
08S540 (BC1F1)
x Rio Grande
x Rio Grande
08S575-12 (BC2F1)
09S236 (BC3F2)
08S574-11 (BC2F1)
08S582-51 (BC1F2)
x Rio Grande
x Rio Grande
09S34-4 (BC3F1)
09S33-14 (BC3F1)
09S45-7 (BC1F3)
x Rio Grande
x Rio Grande
x Rio Grande
10S168 # 2-51 (BC4F1)
09S120-21 (BC4F1)
10S167 # 1-82 (BC4F1)
09S237 (BC1F4)
11S51-18 (BC4F2)
09S204 (BC4F2)
11S65 (BC4F2)
13S74-2 (BC4F3)
13S74-7 EE
13S74-1 PP
11S156 (BC4F3)
13S119-2 (BC4F4)
13S117 EE
13S118 PP
13S161 (BC4F5)
13S140-8 (BC4F5)
14S38 (BC4F5)
14S92 (BC4F6)
14S114 (BC4F6)
14S169 (BC4F6)

Fig. S1

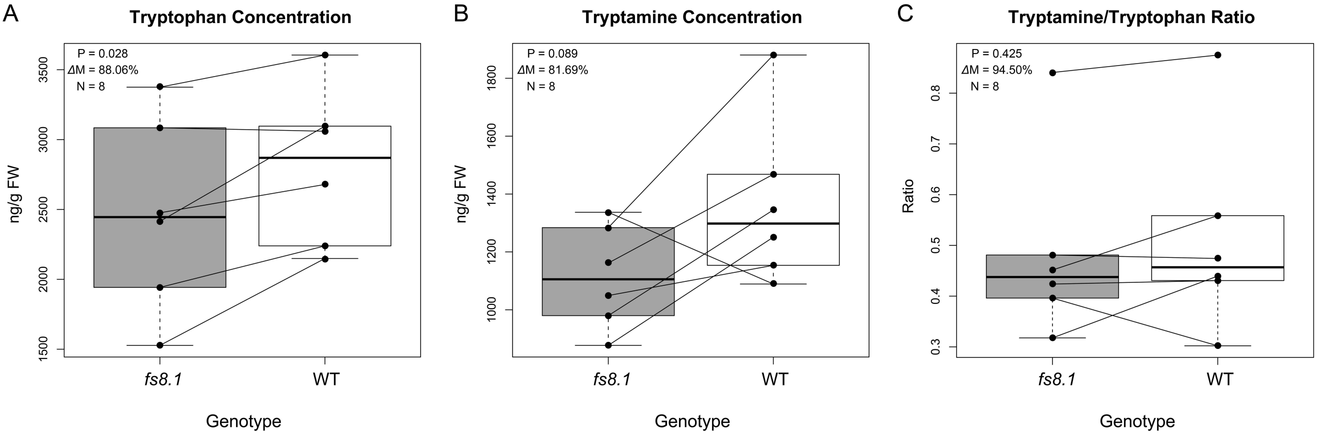
A
Tryptophan Concentration
P = 0.028
ΔM = 88.06%
N = 8
ng/g FW
1500
2000
2500
3000
3500
fs8.1
WT
Genotype
B
Tryptamine Concentration
P = 0.089
ΔM = 81.69%
N = 8
ng/g FW
1000
1200
1400
1600
1800
fs8.1
WT
Genotype
C
Tryptamine/Tryptophan Ratio
P = 0.425
ΔM = 94.50%
N = 8
Ratio
0.3
0.4
0.5
0.6
0.7
0.8
fs8.1
WT
Genotype

Fig. S2
